# High frequency SD-OCT follow-up leading to up to biweekly intravitreal ranibizumab treatment in neovascular age-related macular degeneration

**DOI:** 10.1038/s41598-021-86348-2

**Published:** 2021-03-25

**Authors:** Cengiz Tuerksever, Christian Pruente, Katja Hatz

**Affiliations:** 1 Vista Klinik Binningen, Hauptstrasse 55, 4102 Binningen, Switzerland; 2 Department of Ophthalmology, University of Basel, Basel, Switzerland; 3 Institute of Molecular and Clinical Ophthalmology Basel (IOB), Basel, Switzerland; 4 Faculty of Medicine, University of Basel, Basel, Switzerland

**Keywords:** Biomarkers, Diseases, Medical research, Signs and symptoms

## Abstract

A remarkable proportion of neovascular age-related macular degeneration (nAMD) patients respond rather poorly to ranibizumab treatment, in spite of the minimum 4-week follow-up and treatment interval. Usually, retreatments are based on nAMD activity as evaluated by Spectral-domain Optical coherence Tomography (SD-OCT), biomicroscopic fundus examination and visual acuity changes. In this prospective pilot study, we aimed to study SD-OCT changes in a high-frequent follow-up manner (weekly (month 0–6), biweekly (month 7–12)) throughout the first year, which consequently led to intravitreal ranibizumab being administered up to biweekly. Best corrected visual acuity (BCVA) was already significantly improved at week 2. Central retinal thickness (CRT), intraretinal and subretinal fluid (SRF) were significantly improved from week 1 onwards. Half of the patients showed nAMD activity at week 2 or 3 and received the first retreatment earlier than 4 weeks after baseline injection. In total, 46% of retreatments were already applied 2 or 3 weeks after the previous treatment. Greater range of CRT and SRF fluctuation during follow-up was associated with lower final BCVA. Lower baseline BCVA and better SRF improvement at week 2 was associated with greater BCVA improvement. In conclusion, high-frequency SD-OCT follow-up provided a good option for adapting treatment in nAMD individually.

## Introduction

Age-related macular degeneration (AMD) is the most common cause of irreversible vision impairment in industrialized countries^1,2^. The introduction of anti-vascular endothelial growth factor (anti-VEGF) intravitreal treatment has provided a significant improvement in visual and anatomical prognosis in patients with neovascular AMD (nAMD), and has resulted in significant reduction of AMD-related irreversible vision loss in developed countries^3^. Monthly intravitreal injections of ranibizumab showed to be a safe and effective in treating nAMD^4,5^. Currently, there are three alternative anti-VEGF dosing regimens which are frequently used: (1) fixed monthly injections; (2) Pro Re Nata (PRN) regimen; and (3) treat and extend regimen (TER). In all these treatment regimens, the shortest anti-VEGF injection interval is 4 weeks. However, it seems that the anti-VEGF injection regimen can have an influence on the outcome of treatment in nAMD. It has been shown that more frequent monitoring and treatment are possibly related to better visual acuity in nAMD^6^. Wecker et al. reported an association of higher treatment frequency with improved visual acuity in a real-life nAMD cohort^7^. Yet, a remarkable proportion of nAMD patients responded rather poorly to anti-VEGF treatment, in spite of this short and commonly used treatment interval of 4 weeks^8^. In Comparison of AMD Treatment Trials (CATT), the proportion of eyes without intra- and/or subretinal fluid that received ranibizumab injections at 4 weekly intervals was 45.5%, after 2 years^9^. This implies that more than half of patients who received a 4-weekly ranibizumab treatment regimen showed fluid on OCT. Therefore, for the PRN treated groups in CATT, the proportion of patients with remaining or recurred fluid on OCT was substantially higher (e.g. ranibizumab PRN group 75%) after 2 years^9^. Studies implementing the TER also showed a remarkable proportion of patients in both (treatment naïve and pretreated) populations being continuously treated with 4 weeks intervals due to persistence of fluid on OCT or recurrences under longer intervals^10,11^.

Persistent or recurring intra- or sub-retinal fluid may lead to degenerative changes in the neurosensory retina resulting in reduced long-term visual outcomes^6^. An important question on how to optimize treatment in those eyes is raised. Besides considering combination treatments^12,13^, switching to another anti-VEGF drug^14,15^ or increasing the dose or the injection frequency of the anti-VEGF drug have already been discussed. In the HARBOR Study, increasing the dose of ranibizumab revealed equivalent visual and anatomical outcomes with high dose (2.0 mg) and conventional dose (0.5 mg) in treatment naïve nAMD after 12 and 24 months^16^. Four-weekly 2.0 mg ranibizumab dosing appeared to be safe^16^. Similar visual and anatomical results were shown by Chan et al., with the difference in the higher dose that led to earlier vision improvement and reduction in subretinal fluid and pigment epithelial detachment height^17^. Alternatively, biweekly dosing has been discussed as well. Stewart et al. presented a mathematical model showing that biweekly anti-VEGF dosing might even be a better option than the 4-weekly high dosing regimen^18^. Unfortunately, so far there is very limited information on biweekly anti-VEGF treatment available. Two recently published retrospective case series showed favorable results in either alternating ranibizumab/bevacizumab or bevacizumab biweekly dosing without safety concerns despite the fact that they were not powered for a safety analysis^19,20^.

In addition, the recent improvements in OCT technologies with almost micron precision imaging and introduction of SD-OCT based follow-up within the majority of retina departments enable a novel insight into retinal pathologies^6^. The follow-up mode and eye tracking systems allow the detection of very small alterations in retinal structures in SD-OCT scans at exactly the same position for each visit and the discovery of morphological predictors for anti-VEGF treatment outcomes^6^. If the goal is to evaluate the potential benefit of high frequency anti-VEGF treatment, then follow-up examinations including SD- OCT imaging might be needed earlier than 4 weeks after the last injection. Novais et al. reported a daily SD-OCT follow-up performed during 30 days after the first bevacizumab injection^21^. In this prospective, interventional study of 9 eyes, in approximately half of the patients the individual minimal central retinal thickness was found to be between day 14 and 17 with an increase of fluid afterwards^21^. This supports the approach of biweekly injections if needed. Unfortunately, there was no high-frequency OCT follow-up data after 30 days available. It remains unclear, whether the course of OCT changes followed the same pattern after initial high-frequency treatment in those eyes.

Therefore, this study was designed to evaluate SD-OCT changes in a high-frequent follow-up manner throughout the first year, which consequently led to intravitreal ranibizumab retreatment being administered earlier than 4 weeks after the last treatment (up to biweekly) in nAMD. SD-OCT changes, efficacy and outcome predictors were determined; any safety issues were reported despite this pilot study not being powered for statistical safety analysis.

## Materials and methods

This 12-month prospective, investigator initiated single center, pilot study was conducted in patients with angiographically confirmed treatment naive nAMD at Vista Klinik Binningen, Switzerland (data collection was done between 2011 and 2016, followed by data/image processing between 2017 and 2019). The study was performed in accordance with the principles of Helsinki and good clinical practice (ICH GCP E6 until 2016 and E6(R2) from 2017 onwards). Approval for this pilot study was obtained from the local ethics committee (Ethikkommission Nordwestschweiz, EKNZ, Reference No EKNZ 290/09) and the Swiss health authority (Swissmedic, Reference No 2010DR4241, approval 12/2010). This trial has been registered at ClinicalTrials.gov (NCT03393767, first submitted 02/2011, by ClinicalTrials.gov submission accepted (first posted date) 01/2018). Patients signed an informed consent before enrollment in the study. The primary endpoint of this study was to evaluate SD-OCT changes in a high-frequent follow-up manner throughout the first year, particularly to determine the time point (in relation to the last treatment) of appearance of nAMD activity-defining SD-OCT parameters such as sub- or intraretinal fluid.

Inclusion and Exclusion criteria were adapted to those of ANCHOR/MARINA^4,5^, with the exception of extending the baseline visual acuity range up to 20/32 (78 letters) instead of 20/40 (lower limit 20/320). All patients were treatment naïve with active and newly diagnosed nAMD by both SD-OCT and fluorescein angiography regardless of the choroidal neovascularisation (CNV) subtype. Only one eye of each patient recruited was treated according to the protocol. In case of bilateral newly diagnosed and in-/exclusion criteria fulfilling CNV, the eye with the least fibrosis was chosen as the study eye. In total, 37 follow-up examinations over a period of 12 months were performed for each patient; first on a weekly basis (0–6 month) which was extended to biweekly (7–12 month) follow-up examination visits thereafter. Follow-up visits typically included best-corrected visual acuity measurement (BCVA) following a 4 m ETDRS protocol refraction, biomicroscopic fundus examination and SD-OCT imaging at each visit. SD- OCT scans (Spectralis, Heidelberg Engineering, Heidelberg, Germany) were acquired using an established protocol comprising 19 × 6-mm horizontal scans (volume scan) in a follow-up modus (high-resolution modus, 9 frames, 512 A-scans) and the 6-mm star scan (high-resolution modus, 9 frames, 512 A-scans). All intravitreal injections of 0.5 mg ranibizumab (Lucentis, Novartis, Basel, Switzerland) were applied according to a standard procedure^22^ at baseline (single injection, no loading phase) and afterwards up to 2-weekly according to the following predefined retreatment criteria: persistent or new intra-(IRF) or sub-retinal fluid (SRF) on SD-OCT or new nAMD-related hemorrhage formation on biomicroscopic fundus examination. Retreatments were not possible earlier than 2 weeks after the last treatment, even in case of persisting activity on SD-OCT. Retreatments earlier than 4 weeks after the last treatment (after 2 or 3 weeks) were considered as “early retreatments”.

In this study, SD-OCT scans were analyzed by the same observer (C.T.) following standard evaluation protocols for central point choroidal thickness (ChT), central retinal thickness (CRT) (1 mm central subfield thickness), number of hyperreflective foci (HRF), integrity of photoreceptor inner/outer segment (IS/OS) junction line/extern limiting membrane (ELM), sub- and intraretinal fluid (SRF, IRF) characteristics within the central 1 mm ETDRS subfield and vitreomacular interface status. For SRF and IRF the maximum height of SRF and biggest cysts were measured, respectively. Fluctuation of CRT/SRF was defined as the difference of the maximum and minimum value during follow-up period (excluding baseline value). IS/OS junction line and ELM integrity was evaluated as described by Hatz et al.^23^ as the mean of the horizontal and vertical SD-OCT scans and graded as: 0 (no disruption in 1-mm center), 1 (mild disruption < 1/4 within 1-mm center), 2 (1/4 to 3/4 disruption within 1-mm center), and 3 (> 3/4 disruption within 1-mm center). RPE atrophy in fundus autofluorescence images and size of leakage of CNV in fluorescein angiography were recorded at the baseline and exit visit.

SPSS version 23.0 (IBM SPSS, Chicago, IL, USA) was used for statistical analysis. All values are reported as mean ± standard deviation. A two-sided unpaired t-test was used to compare differences between subgroups. A two-sided paired t-test was used to compare differences from the baseline to follow-up. Pearson correlation was used to analyze the relationship between baseline demographics (like age), morphological measurements (like number of HRF) and outcome parameters like numbers of treatment and visual acuity. The level of statistical significance was set at 0.05.

## Results

For this study, we included a total of 24 eyes of 24 patients with treatment naive nAMD. One patient prematurely withdrew consent less than 3 months after inclusion due to the burden of weekly assessments. The remaining 23 (female/male = 16/7) patients completed the study and were included in the efficacy analysis. At baseline, the mean age was 82.3 (± 6.8) years. All patients were recruited from the Medical Retina Department of Vista Klinik Binningen, Switzerland and treated according to the study protocol between 10/2011 and 08/2016. For detailed patient demographics and baseline characteristics see Table 1. Fluoresceine angiography at baseline revealed 16 (69.6%) type 1, 6 (26.1%) type 2 and 1 (4.3%) type 3 choroidal neovascularisation. Five patients (21.7%) presented with mild sub-retinal hemorrhage at baseline. In these five cases, analysis of SD-OCT images was not affected by the hemorrhages.

**Table 1:** Patient demographics and baseline characteristics (n = 23).

	Mean	± SD	Range
Gender: M/F, n (%)	7 (30.4)/16 (69.6)		
Age, years	82.3	6.8	66–93
N ranibizumab injections	7.86	5.06	1–19
CRT, µm	418.7	132.6	289–757
BCVA, ETDRS letters	67.8	9.58	39–78
Morphological finding	Proportion, n (%)		
PVD	17 (73.9)		
RPE atrophy	9 (39.1)		
SR hemorrhage	5 (21.7)		
IS/OS line disruption	20(86.9)		
ELM disruption	19(82.6)		

*F* female, *M* male, *N* number, *NA* not applicable, *SD* standard deviation, *CRT* central retinal thickness, *BCVA* best-corrected visual acuity, *ETDRS* Early Treatment Diabetic Retinopathy Study, *PVD* posterior vitreous detachment, *RPE* retinal pigment epithelium, *SR* sub-retinal, *IS/OS* photoreceptor inner/outer segment (IS/OS) junction line, *ELM* external limiting membrane.

### BCVA efficacy

**Figure 1:**
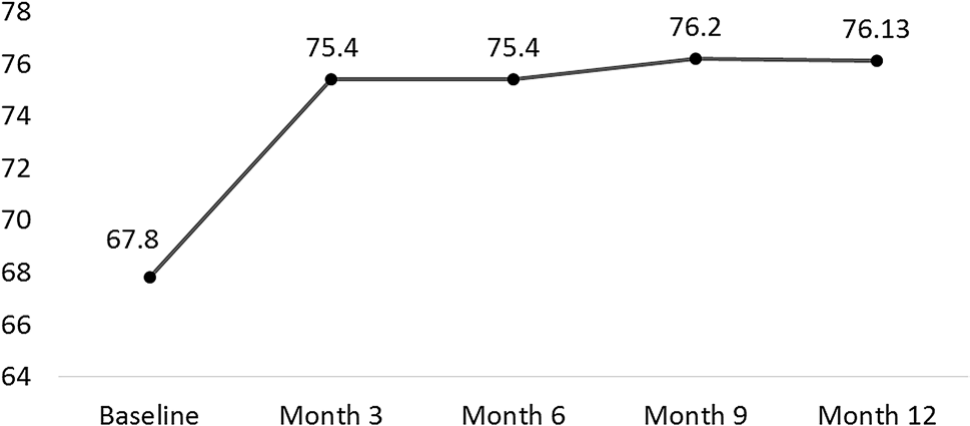
Mean BCVA (ETDRS Letters) during 12 months follow-up.

Improvement in mean BCVA score from baseline (67.8 ± 6.8 letters) already reached significant levels at week 2 (70.3 ± 12.2 letters, *p* = 0.048) and for all other follow-up examinations. Mean BCVA score significantly improved from baseline to month 12 (76.1 ± 15.6 letters, *p* = 0.013). For mean BCVA follow-up see Fig. 1. Twenty two (96%) of 23 patients had a BCVA improvement of at least one letter at month 12 compared to baseline. Eighteen eyes (78%) had a BCVA gain of ≥ 5 letters and 11 eyes (48%) ≥ 10 letters, at month 12. One eye had a BCVA loss of ≥ 25 letters due to an extensive sub-retinal hemorrhage. Furthermore, the patients were grouped according to their baseline BCVA (< 69 and ≥ 69 Letters; 69 letters for overall median BL BCVA). Mean BCVA gain at month 12, was significantly higher for eyes with lower baseline BCVA (n = 11, 14.9 ± 7.9 letters) than for eyes with higher baseline BCVA (n = 12, 2.3 ± 17.1 letters, *p* = 0.017).

### High-frequency SD-OCT follow-up leading to early retreatment

After baseline injections (n = 23), 158 retreatments were applied due to activity on SD-OCT. No reactivation only due to hemorrhage without SD-OCT reactivation signs was noticed. Within 12 months, a total of 181 treatments were carried out. All eyes received a mean of 7.9 ± 5.1 (range 1–19) ranibizumab injections. Only four (17.4%) eyes received more than 12 injections in total during the 12 months follow-up period; but 15 eyes (65%) showed at least once early/persisting activity on SD-OCT at week 2 or 3 after the last treatment leading to retreatment < 4 weeks after previous treatment (early retreatment). Due to activity on SD-OCT, out of all 158 retreatments, 73 (46%) were already applied 2 or 3 weeks after the previous treatment (early retreatment).

**Figure 2:**
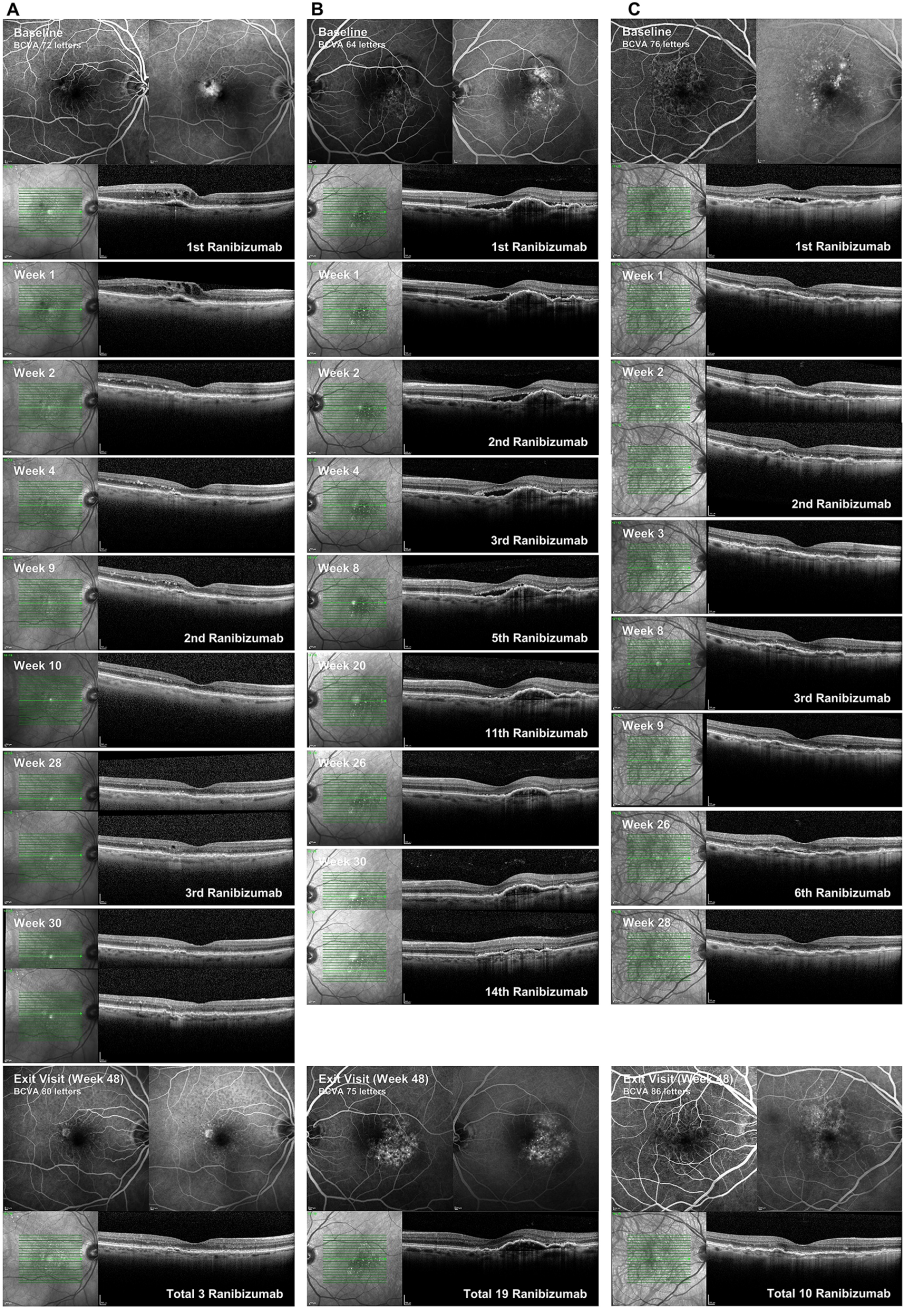
Examples of different activation/reactivation patterns (first line—baseline findings on fluoresceine angiography (FA) early and late phase and SD-OCT; last line—exit visit (48 weeks) FA early and late phase and SD-OCT; in between selected SD-OCT follow-up scans): (**A**) Type 3 CNV with only two reactivations during 12 months follow-up, no persisting fluid/early reactivation 2 or 3 weeks after last treatment and therefore never fulfilling the criteria for early retreatment, total 3 injections, BCVA gain 8 letters. (**B**) Type 1 CNV with persisting subretinal fluid during the first months of treatment and therefore, several times fulfilling the criteria for early retreatment, after reaching dryness several recurrences, total 19 injections/ xx out of these earlier than 4 weeks after last treatment (early retreatment), BCVA gain 11 letters. (**C**) Type 1 CNV with a small amount of persisting subretinal fluid at week 1 and 2 and therefore, fulfilling the criteria for early retreatment at week 2, afterwards recurrences at intervals of minimum 4 weeks, total 10 injections/ 1 out of these earlier than 4 weeks after last treatment (early retreatment), BCVA gain 10 letters.

One patient (4%) did not experience any reactivation on SD-OCT which would have induced retreatment after the first baseline injection within 12 months (small type 2 CNV). For the remaining 22 patients, the mean interval from baseline injection to first retreatment was 6.8 ± 8.5 weeks (range 2–39 weeks). Out of 23 patients, 11 (48%) showed persisting activity on SD-OCT and received the first retreatment already 2 weeks after the baseline injection, with only one patient (4%) showing a reactivation and receiving treatment after 3 weeks. In total 12 of 23 patients (52%) received the first retreatment before the end of the first four weeks due to persistent or early recurring intra- or sub-retinal fluid on SD-OCT. There was a trend for younger patients requiring less retreatments compared to elderly patients (*r* = − 0.411, *p* = 0.052) who needed treatment more frequently. For examples of different activation/reactivation patterns on SD-OCT see Fig. 2.

### Further morphological changes on high-frequency SD-OCT follow-up and FA

**Figure 3:**
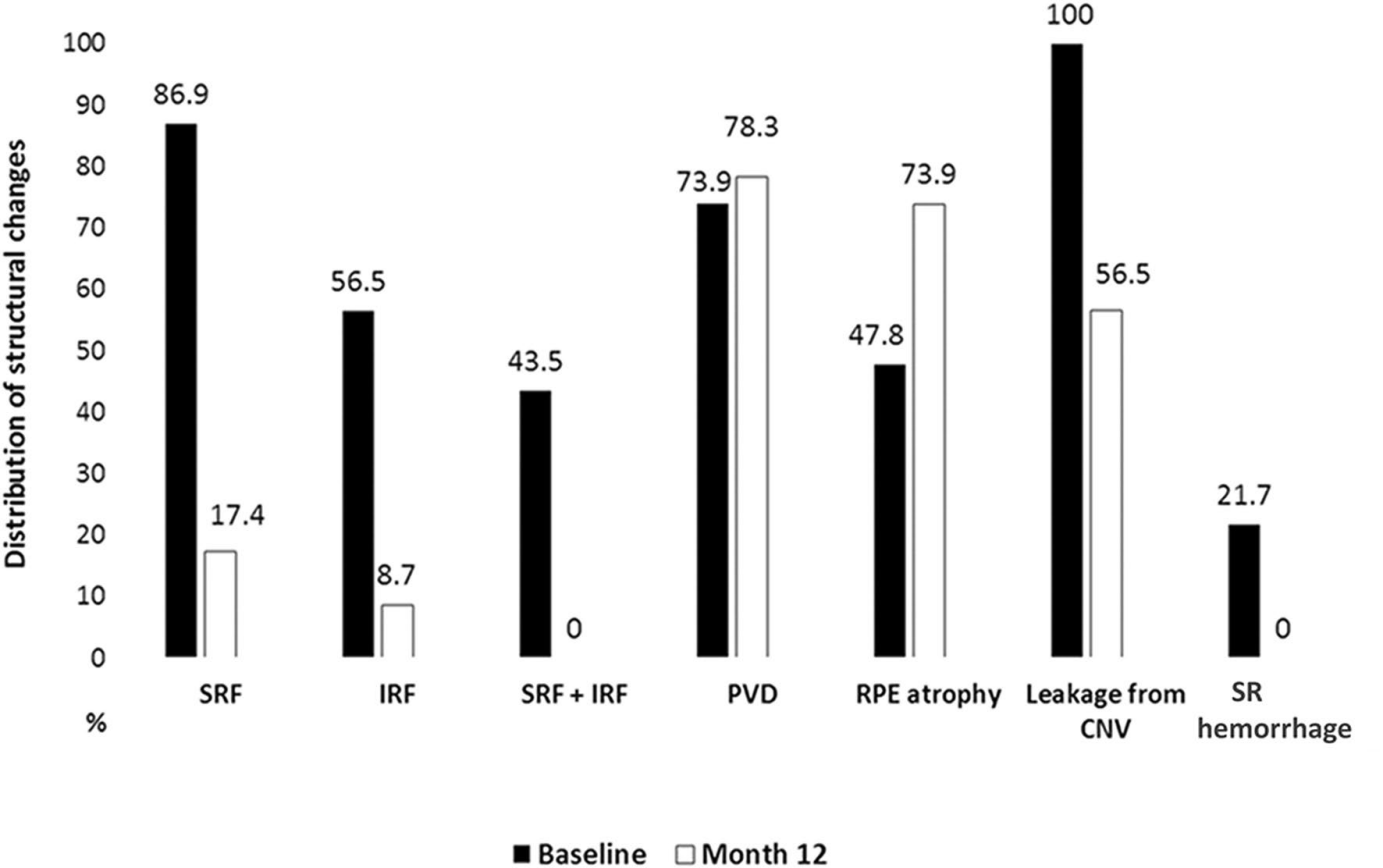
Proportion of patients (%) with key structural findings at baseline and month 12. SRF, subretinal fluid; IRF, intraretinal fluid; PVD, posterior vitreous detachment; RPE atrophy, Retinal pigment epithelial atrophy; Leakage from CNV, leakage from Choroidal Neovascularisation; SR hemorrhage, subretinal hemorrhage.

High-frequency SD-OCT follow-up showed that mean CRT, IRF and SRF height were already significantly improved (*p* = 0.0001; *p* = 0.014; *p* = 0.003) at week 1 and improvements remained stable during 12 months (Table 2). Mean CRT change was significantly higher after first treatment than for any other subsequent injections. Mean number of hyperreflective foci (HRF) was significantly reduced from week 2 (*p* = 0.002) onwards until the end of month 12 (Table 2). High-frequency SD-OCT follow-up revealed SRF in 20 (86.9%) eyes at baseline, 15 (65.2%) at week 1, 11 (47.8%) at week 2, 8 (34.7%) at week 3, 5 (21.7%) at week 4, 6 (26.1%) at week 6, 3 (13.0%) at week 8 and between 3 and 7 (13.0–30.4%) for following visits, at 12 months 4 (17.4%). In contrast to SRF, IRF was present in 13 eyes (56.5%) at baseline and showed a quicker reduction to 3 (13.0%) at week 1 and 2 and 3 (in the same eyes), 2 (8.7%) at week 4, 1 (4.3%) at week 6, 2 (8.7%) at week 8 and between 0 and 3 (0–13.3%) for the following visits; at month 12 2 (8.7%). Nearly half of the eyes (10 (43.5%)) showed both, SRF and IRF, at baseline. At month 12, we did not observe either of, SRF and IRF in the same eye. Proportion of patients (%) with key structural findings at baseline and month 12 are shown in Fig. 3. Presence of CNV leakage was reduced from 100% to 56.5% at month 12. Mean size of CNV leakage was significantly reduced from 1.95 ± 2.03 mm^2^ at baseline to 0.971 ± 1.65 mm^2^ at month 12 (*p* = 0.0001). We observed a non-significant reduction in mean central point choroidal thickness from 168.5 ± 64.05 µm to 161.9 ± 65.9 µm (*p* = 0.091) at month 12.

**Table 2:** SD-OCT based short- and long-term structural improvement.

	Mean, (SD), *p*-value (t-test, compared with baseline)					
	Baseline	Week 1	Week 2	Week 4	Month 3	Month 12
CRT, µ	418.7 (132.6)	328.6 (67.1) *P* < 0.0001	308.6 62.9) *P* < 0.0001	290.3 55.7) *P* < 0.0001	283.5 (43.7) *P* < 0.0001	288.6 (47.8) *P* < 0.0001
IRF, µ	71.74 (114)	12.5 (35) *p* = 0.014	1.35 (6.5) *p* = 0.007	1.78 (8.5) *p* = 0.007	0.74 (3.54) *p* = 0.006	3.04 (10.8) = 0.007
SRF, µ	113.17 (93.9)	64.8 (64.6) *p* = 0.003	42 (62.4) *P* < 0.0001	24.35 (48.3) *P* < 0.0001	7.91 (24.9) *P* < 0.0001	9.1 (21.5) *P* < 0.0001
ChT, µ	168.5 64)	162 (66.1) *p* = 0.093	160.5 (63.7) *p* = 0.052	162.6 (61.4) *p* = 0.089	161.6 (62.6) *p* = 0.019	162 (66) *P* = 0.091
HRF, N	12.5 (6.6)	11 (5.8) *p* = 0.26	8.4 (5) *p* = 0.002	6.7 (4.25) *P* < 0.0001	6.96 (5.5) *p* = 0.001	4.3 (3.3) *P* < 0.0001

*CRT* central retinal thickness, *IRF* maximum height of intraretinal fluid, *SRF* maximum height of central subretinal fluid, *Cht* central point choroidal thickness, *HRF* hyperreflective foci at central 1 mm diameter.

Posterior vitreous detachment was present in 73.9% (17) of eyes at baseline and 78.3% (18) at month 12. This was not related to the number of the injections (*r* = − 0.005; *p* = 0.813).

### Morphological outcome predictors

Both, baseline integrity of IS/OS line and external limiting membrane (ELM), indicated better baseline BCVA (*p* = 0.006; *p* = 0.006). Eyes with less ELM disruption at baseline (grade 0 and 1) showed higher BCVA improvement at 12 months than eyes with more ELM disruption (grade 2 and 3) (*p* = 0.029). The number of hyperreflective foci (HRF) at baseline was correlated with the IS/OS line disruption score at baseline (*r* = 0.437; *p* = 0.037), meaning that eyes with more HRF at baseline showed increased IS/OS line disruption at baseline. High-frequency SD-OCT showed that a rapid reduction in HRF at week 2 was associated with lower CRT (*p* = 0.024) at month 12. Eyes with more HRF at baseline showed significantly more reduction in CNV leakage area at Month 12 (*p* = 0.023). A rapid improvement in HRF at week 2 indicated a smaller CNV leakage area at month 12 (*p* = 0.007). Greater range of fluctuation of CRT and SRF during 12 months led to lower BCVA (*p* = 0.006, *p* = 0.005, respectively) at month 12. A rapid improvement in SRF at week 2 predicted better BCVA improvement (*r* = 0.547, *p* = 0.007) at month 12. The eyes (11/23) in which the maximum SRF height was reduced to 0 (meaning complete resolution of SRF) at week 2 showed the following FA based CNV classifications: 7/11 (64%) type 1 and 4/11 (36%) type 2 CNV. Further two eyes had no SRF at baseline and week 2 (1 × type 3, see Fig. 2A; 1 × type 1). The eleven eyes with complete resolution of SRF at week 2 had the same mean CNV leakage area at baseline compared to those without complete resolution (2.123 ± 0.769 vs. 2.084 ± 0.471 mm^2^, *p* = 0.963).

Eyes with more SRF at baseline received more often early retreatments (earlier than 4 weeks after the previous treatment) compared with those with less SRF at baseline (*p* = 0.014). However, presence of IRF at baseline was not related to high frequency treatment (*p* = 0.343).

### Safety

**Figure 4:**
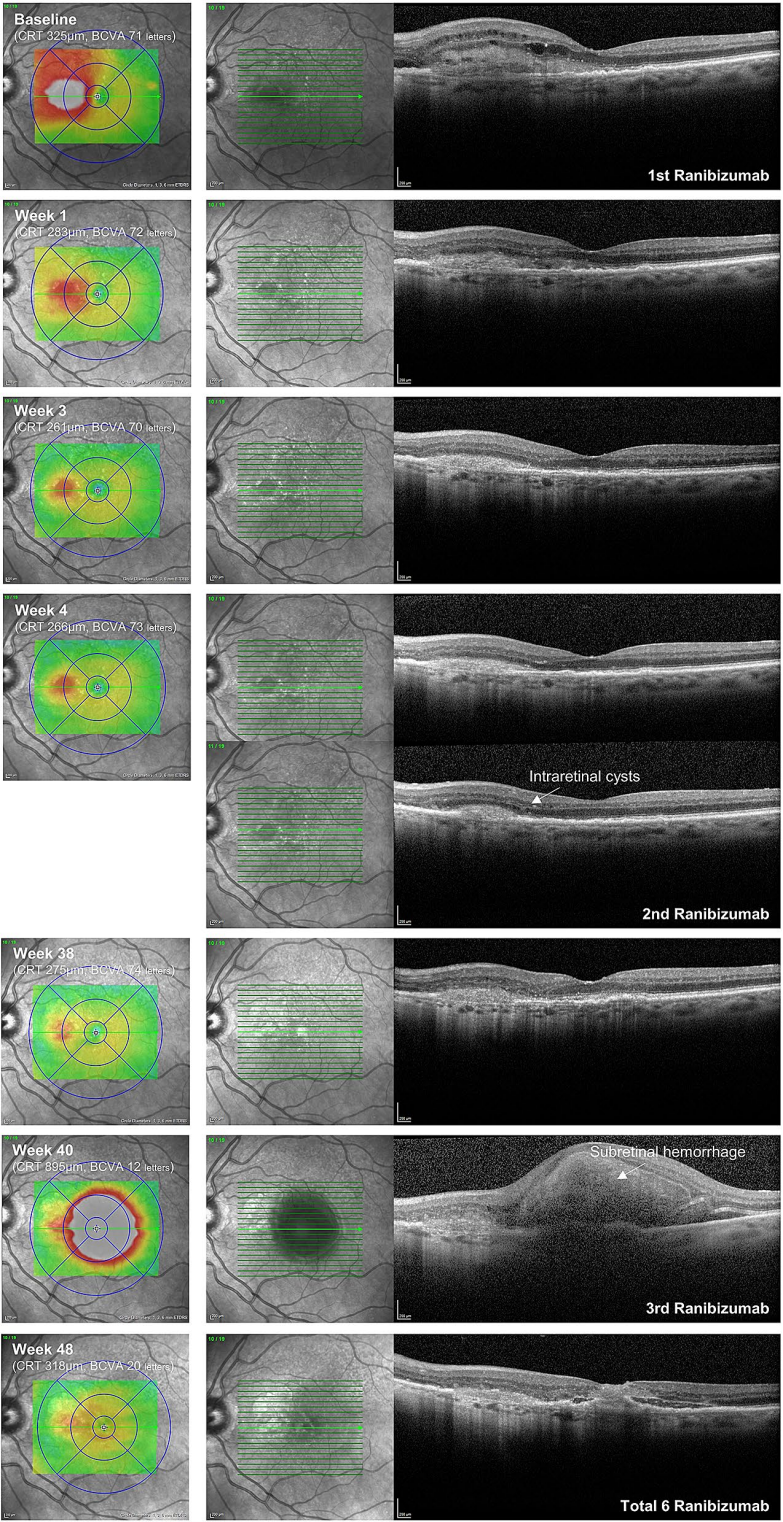
Baseline and selected follow-up SD-OCT scans of a patient having experienced an extensive subretinal hemorrhage at week 40. Left column – thickness map, right column – SD-OCT section.

As this pilot study with the primary aim of observing high-frequency SD-OCT changes not being powered for a statistical safety analysis in terms of treatment severe adverse are only reported as a case description. During the 12 months follow-up, one vision threatening adverse event occurred. A detailed SD-OCT follow-up of this case is shown in Fig. 4. The eye presented with a new extensive subretinal hemorrhage at Month 10; the previous injection took place 36 weeks earlier, and therefore is not likely to be related to this event. This patient received 2 injections (first at baseline and again at week 4) within the follow-up period until the adverse event occurred (meaning that the patient did not receive any early retreatment prior to the adverse event). The last SD-OCT was performed 2 weeks before to the adverse event happened. No clear sub- or intraretinal fluid or hemorrhage have been detected 2 weeks before the event and therefore, the retreatment criteria were not fulfilled. A retrospective careful review of the SD-OCT volume scan sections showed a very discreet amount of newly developed subretinal hyperreflective material (SHRM). The sub-retinal hemorrhage, which occurred 2 weeks later, led to a severe reduction in BCVA (− 51 letters at month 12 compared to baseline (20 vs. 71 letters)). After the hemorrhage (week 40) the patient received bi-weekly ranibizumab injections up to regular study exit at week 48.

No other severe adverse events occurred during follow-up.

## Discussion

In this prospective study we investigated a high-frequency SD-OCT follow-up which could lead to early (up to biweekly) intravitreal ranibizumab retreatment in patients with nAMD. Imaging and other baseline biomarkers as outcome correlating factors were evaluated.

In our study, during the entire follow-up period, due to activity on high-frequency SD-OCT follow-up 46% of all retreatments were already applied 2 to 3 weeks after the previous treatment and 65% of eyes showed at least once early/persisting activity on SD-OCT at week 2 or 3 after the last treatment. This seems to correspond with the findings from CATT where the proportion of eyes without intra- and/or subretinal fluid was only 45.5% at the 2 year exit visit (4 weeks after the last ranibizumab injection)^24^, meaning that more than half of patients showed fluid on OCT despite 4-week treatment intervals and at least a few of them could have fulfilled our early retreatment criteria. Recently, Real et al. evaluated the literature for the reactivation time in the PRN nAMD treatment and calculated that 50% of patients presented a reactivation before 70 days after the last injection^25^ with detection of recurrence even being delayed in some cases by 2–3 weeks in real-life analyses compared to prospective studies. However, none of the included studies reported a follow-up period shorter than monthly intervals. Therefore, we assume that more frequent follow-up could have led to a shorter treatment interval. But it remains unclear if this would have translated to better outcomes. Our study population showed a mean treatment interval from baseline injection to first retreatment of 6.8 ± 8.5 weeks (≈48 days) ranging between 2 and 39 weeks. This reflects the uniqueness of follow-up and treatment regimens for nAMD which needs to be addressed by using individualized treatment schemes.

Despite a total number of treatments which is comparable to previous studies using less intensive follow-up regimen, 65% of eyes showed at least once early/ persisting activity on SD-OCT at week 2 or 3 after the last treatment leading to retreatment earlier than 4 weeks after previous treatment in this study. The need for early retreatment might be even more relevant in the beginning of treatment since more than half of our patients (52%) showed persisting/recurring SD-OCT activity at 2 or 3 weeks after baseline and therefore, received the first retreatment earlier than 4 weeks after baseline injection. Chan et al. reported that a higher dose of 2.0 mg ranibizumab led to earlier vision improvement and reduction in subretinal fluid and pigment epithelial detachment height^17^. Increasing the dose might cause other effects than increasing the frequency of the normal dose. This arises the question of whether an early retreatment especially in the beginning could speed up the resolution of edema in cases with delayed or poor response under regular 4-week intervals. In our study mean CRT, IRF and SRF height were already significantly improved at week 1 but 48% of eyes showed persisting amounts of SRF or IRF at week 2. This poses the question if a high-frequency "loading" would make a difference for these cases. Fig. 2B illustrates a case of incomplete SRF resolution in the beginning of treatment and therefore, receiving multiple biweekly injections and reaching dryness under this therapy. The example in Fig. 2C shows a small amount of persisting SRF at week 1 and 2 leading to early retreatment, too, but later on retreatments in ≥ 4 weeks intervals. In our study, visual acuity improvement subsequently followed morphological improvements by reaching significance at week two and even further but it remains unclear if early retreatment had an impact on that, especially as the mean visual acuity gain was comparable to previous PRN studies with less intense SD-OCT follow-up. However, even with a very intense follow-up mode severe vision loss due to an extensive hemorrhage can happen. It seems crucial to include not only SRF, IRF and newly developed hemorrhage but also other criteria like subretinal hyperreflective material in the activity analysis.

Two recently published retrospective case series showed favorable results in either alternating ranibizumab/bevacizumab or bevacizumab biweekly dosing without new safety signals, despite not being powered for a statistical safety analysis^19,20^. Witkin et al. reported significant VA improvement as well as CRT and pigment epithelial detachment height to have decreased after four biweekly alternating ranibizumab/bevacizumab injections^19^. Mimouni et al. found improvement or complete resolution of subretinal fluid in about one third of anti-VEGF bad responders treated with 3 to 4 biweekly bevacizumab injections^20^. Unfortunately, both retrospective case series only evaluated a very short time period of up to 8 weeks and did not include high-frequency OCT follow-up in parallel. In our study within a prospective follow-up of 12 months, no adverse events which were likely to be related to retreatments applied earlier than 4 weeks after previous treatment (early retreatment) were observed. However, as a pilot study with the primary aim of observing high-frequency SD-OCT changes our study was not powered for a statistical safety analysis. At least for systemic adverse events we know that for cancer treatment systemic anti-VEGF therapy (like bevacizumab) is applied on a biweekly basis with an acceptable safety profile.

Similar to CATT^26^, in our study the evaluation of outcome correlating factors revealed that eyes with lower baseline visual acuity gained more than eyes with higher baseline visual acuity. Baseline integrity of IS/OS line and external limiting membrane (ELM) indicated better baseline BCVA. It has been reported by several authors that there is a significant association between macular atrophy as expressed by the condition of the RPE, IS/OS junction, ELM and visual acuity in AMD^27–29^. In our study the high-frequency SD-OCT follow-up revealed that a rapid improvement in subretinal fluid (SRF) at week 2 was associated with better BCVA improvement at Month 12. While intraretinal fluid (IRF) seemed to be associated with visual impairment due to loss of neurosensory retina in nAMD^6^, it was controversially discussed whether the presence of SRF can at least have a negative influence on the visual outcome over a longer term^30^ or be tolerated as potentially beneficial^31^. On the basis of our results, we cannot make assumptions on the protective effect of SRF. At least more rapid resolution of intraretinal fluid, and therefore reduction in CRT, seems to predict good visual outcomes^32^ and might also be an advantage of higher frequent OCT follow-up with an early anti-VEGF retreatment. Further, higher frequent OCT monitoring followed by an immediate retreatment if necessary might prevent greater fluctuation of CRT and SRF, as defined by the difference of maximum and minimum value during follow-up (excluding baseline value). In our study, greater range of fluctuation of CRT and SRF during the follow up period led to lower BCVA at month 12.

In our study the number of hyperreflective foci (HRF) on SD-OCT was significantly decreased from week two onwards throughout to month 12. HRF have been hypothesized as a clinical biomarker of inflammatory response, aggregates of microglia activated cells, protein and/or lipid deposits, lipid-laden macrophages and migrated retinal pigment epithelial cells^6,33–35^. In our patients, presence of more HRFs at SD-OCT scans at baseline was associated with increased IS/OS line disruption at baseline. This might express a more progressed nAMD in itself but due to more severe blood–retina barrier damage, eyes with more HRF at baseline might also have more active inflammation followed by loss of IS/OS line integrity due to inflammatory response to/within the outer retina. Coscas et al.^33^ reported resolution of HRF being associated with better final BCVA and Hsia et al.^36^ concluded that eyes with reduced HRF in the subretinal space at 3 months having better visual improvement at 12 months. Segal et al. found a greater CRT reduction in eyes with rapid reduction of HRF^37^. This interesting finding was confirmed by our results. Due to lack of comparison to a regular treated control group it remains unclear if early anti-VEGF retreatment might have supported a rapid reduction of HRF.

To the best of our knowledge, this is the first prospective study evaluating SD-OCT changes in a high-frequent follow-up manner of 1 year, resulting in an early (up to biweekly) intravitreal ranibizumab retreatment in nAMD. Strengths of the study are its prospective nature as well as its consequent and tight follow-up mode. The limitation is the lack of a control group with the same follow-up mode and assessment scheme of only up to 4-weekly treatments (regular label treatment). Without a control group we cannot make a determination if early retreatment after 2 or 3 weeks might have an impact on visual acuity outcome compared to conventional treatment. The decision against such a control group was made because of the observational character of the primary issue of this study in terms of evaluation of SD-OCT changes and further the awaited slow recruitment due to the high frequent study visits being a high burden on the patients.

In conclusion, high-frequency SD-OCT follow-up might provide a good option for individualizing treatment in nAMD, possibly with early retreatment aiming to reach earlier and—if necessary—stronger treatment response. The results of this pilot study need to be confirmed in larger scale studies. The discovered outcome predictors might provide guidance for an efficient treatment plan regarding disease management.
